# Prevalence of Risk Factors Among Patients With Glaucoma in Jeddah, Saudi Arabia

**DOI:** 10.7759/cureus.37689

**Published:** 2023-04-17

**Authors:** Ahmed M Al-Manjoumi, Majed A Filimban, Mohammed S Bantan, Hussam A Abualhamayel, Omar A Youldash

**Affiliations:** 1 Ophthalmology, Faculty of Medicine, King Abdulaziz University, Jeddah, SAU; 2 General Practice, Faculty of Medicine, King Abdulaziz University, Jeddah, SAU

**Keywords:** risk factors, primary open-angle glaucoma, prevalence, primary congenital glaucoma, angle closure glaucoma

## Abstract

Objective

The objective of this study is to estimate the prevalence of risk factors among patients with glaucoma in Jeddah, Saudi Arabia.

Methods

This cross-sectional study was conducted on 215 patients diagnosed with glaucoma in the period between March 2022 and August 2022 at King Abdulaziz University Hospital, in Jeddah, Saudi Arabia. We used the participants’ medical records and contacted the patients to collect information on sociodemographic characteristics and known risk factors of glaucoma.

Results

Among the 215 patients with glaucoma, 142 (66.0%) patients had open-angle glaucoma, 15 (7.0%) patients had closed-angle glaucoma, and 58 (27.0%) patients had congenital glaucoma. Among the patients with open-angle glaucoma, 122 (85.9%) patients were aged > 40 years, and 99 (69.7%) patients had myopia. Among the patients with closed-angle glaucoma, 13 (86.7%) patients had hyperopia and 10 (66.7%) patients were aged > 60 years. Among the patients with congenital glaucoma, 21 (36.2%) patients had a family history of congenital glaucoma and 28 (48.3%) patients had consanguine parents.

Conclusion

The prevalence of advanced age, hyperopia, and consanguine parents was the highest among patients with open-angle glaucoma, closed-angle glaucoma, and congenital glaucoma, respectively. These findings could inform public health policies among practitioners involved in ophthalmological care.

## Introduction

The Greek term glaukos describes diseased eyes and was translated into Zarqaa in the Arabic Middle Ages [1]. In 1850, the term ophthalmoscope was introduced, after its invention by German physician Hermann von Helmholtz, allowing visualizing the optic nerve and observing eye diseases [2-4]. Glaucoma refers to a heterogeneous group of ocular disorders characterized by intraocular pressure (IOP)-associated optic neuropathy that can cause optic nerve damage and subsequent complete visual loss [5]. Worldwide, glaucoma is considered the second leading cause of blindness [6]. There are several types of glaucoma, with the two major types being primary open-angle glaucoma (POAG) and angle-closure glaucoma (ACG) [7]. POAG is characterized by chronic, asymptomatic, progressive, and bilateral loss of optic nerve, which often occurs owing to increased IOP [8]. In contrast, ACG is associated with increased IOP due to aqueous outflow obstruction resulting in optic nerve damage [9]. There are numerous risk factors for glaucoma, including increased IOP, age > 40 years, diabetes mellitus, systolic hypertension, and a family history of glaucoma [10]. A 2019 study conducted in the Riyadh governorate reported that the prevalence of glaucoma was 5.6% [11]. A 2014 population-based study reported that there were 64.3 million patients with glaucoma aged 40-80 years in 2013, which was expected to increase to 111.8 million by 2040 [12]. Accordingly, this disease is considered important; however, there have been few Saudi Arabian studies on glaucoma, including investigations into the prevalence of risk factors, which could inform prevention measures. Accordingly, we aimed to estimate the prevalence of risk factors among patients with glaucoma in Jeddah, Kingdom of Saudi Arabia.

## Materials and methods

Study design and participants

This cross-sectional study was conducted on 215 patients diagnosed with glaucoma in the period between March 2022 and August 2022 at King Abdulaziz University Hospital (KAUH), a tertiary center in Jeddah, Kingdom of Saudi Arabia. We included patients diagnosed with glaucoma at KAUH, male or female, who lived in Jeddah city and excluded patients only with suspected or unconfirmed glaucoma.

Data collection

We used the participants’ medical records and contacted the patients to collect information on sociodemographic characteristics (such as age group, sex, nationality, marital status, occupational status, and ethnicity) and known risk factors of glaucoma (such as history of high IOP, diabetes mellitus, hypothyroidism, hyperopia, myopia, consanguinity, family history of glaucoma, history of drug use, history of previous vitreoretinal surgery, uveitis, tumors, congenital ocular abnormalities, systemic congenital abnormalities, history of eye injury, rubeosis iridis, and experience of stress/fear).

Ethical considerations

Ethical approval for this study was obtained from the Research Ethics Committee of KAUH, faculty of medicine (Reference No. 178-22). Verbal consent was obtained from all participants.

Statistical analysis

Statistical analyses were performed using IBM SPSS Statistics for Windows, Version 23 (Released 2015; IBM Corp., Armonk, New York, United States). Data are expressed as counts (%). Between-group comparisons were performed using Pearson’s chi-square test. Statistical significance was set at P-values of < 0.05.

## Results

Table 1 shows the demographic characteristics of all patients and subgroups defined by the glaucoma type. Among the 215 included patients, 142 (66.0%) patients had POAG, 15 (7.0%) patients had ACG, and 58 (27.0%) patients had congenital glaucoma. Most patients were aged 19-55 years (n = 54, 25.1%), followed by ≤ 18 years (n = 53, 24.7%), 56-65 years (n = 47, 21.9%), 66-75 years (n = 25, 16.7%), and ≥ 76 years (n = 25, 16.7%). Most patients with POAG were aged 19-55 years (n = 43, 30.3%) and 56-65 years (n = 43, 30.3%), while only five (33.3%) patients with ACG were aged 19-55 years (33.3%). Moreover, most patients with congenital glaucoma were aged ≤ 18 years (n = 52, 89.7%). The proportion of male patients was higher than that of female patients (54.9% vs. 45.1%) among all patients. The proportion of male patients was higher than that of female patients among the patients with POAG and congenital glaucoma, while the reverse was true among the patients with ACG. In all patients and all subgroups, most patients were of Saudi nationality and unemployed. Regarding marital status, there were more married patients among all patients and the patients with POAG and ACG, while most patients with congenital glaucoma were single.

**Table 1:** Demographic characteristics of the patients

Items	All patients (n = 215)	Open-angle glaucoma (n = 142, 66.0%)	Closed-angle glaucoma (n = 15, 7.0%)	Congenital glaucoma (n = 58, 27.0%)
Age category				
≤ 18 years	53 (24.7%)	1 (0.7%)	-	52 (89.7%)
19–55 years	54 (25.1%)	43 (30.3%)	5 (33.3%)	6 (10.3%)
56–65 years	47 (21.9%)	43 (30.3%)	4 (26.7%)	-
66–75 years	25 (16.7%)	21 (14.8%)	4 (26.7%)	-
≥ 76 years	36 (16.7%)	34 (23.9%)	2 (13.3%)	-
Sex				
Male	118 (54.9%)	80 (56.3%)	7 (46.7%)	31 (53.4%)
Female	97 (45.1%)	62 (43.7%)	8 (53.3%)	27 (46.6%)
Nationality				
Saudi	164 (76.3%)	106 (74.6%)	9 (60.0%)	49 (84.5%)
Non-Saudi	51 (23.7%)	36 (25.4%)	6 (40.0%)	9 (15.5%)
Marital status				
Married	146 (67.9%)	129 (90.8%)	13 (86.7%)	4 (6.9%)
Single	64 (29.8%)	10 (7.0%)	-	54 (93.1%)
Widowed	5 (2.3%)	3 (2.1%)	2 (13.3%)	-
Occupational status				
Employed	49 (22.8%)	43 (30.3%)	3 (20.0%)	3 (5.2%)
Unemployed	108 (50.2%)	50 (36.2%)	7 (46.7%)	51 (87.9%)
Retired	49 (22.8%)	45 (31.7%)	4 (26.7%)	-
Students	9 (4.2%)	4 (2.8%)	1 (6.7%)	4 (6.9%)

Among the patients with POAG, 122 (85.9%) patients were aged > 40 years and 99 (69.7%) patients had myopia. Furthermore, a small number of patients with POAG had a family history of glaucoma (21.1%), used topical steroids (20.4%), used oral steroids (15.5%), used intravenous steroids (4.2%), used inhaled steroids (7.7%), and had hypothyroidism (12.0%). There was no significant difference between patients with POAG in terms of increased IOP, a history of diabetes mellitus, and a history of previous vitreoretinal surgery (P > 0.050). Most patients were of Arabic ethnicity (88.0%, P < 0.0001, Table 2).

**Table 2:** Risk factors for open-angle glaucoma (n = 142)

Risk factors	Yes	No	Significance
Age > 40 years	122 (85.9%)	20 (14.1%)	P < 0.0001
High intraocular pressure	82 (57.7%)	60 (42.3%)	P = 0.065
History of diabetes mellitus	80 (56.3%)	62 (43.7%)	P = 0.131
Family history of glaucoma	30 (21.1%)	112 (78.9%)	P < 0.0001
Myopia	99 (69.7%)	43 (30.3%)	P < 0.0001
Use of topical steroids	29 (20.4%)	113 (79.6%)	P < 0.0001
Use of oral steroids	22 (15.5%)	120 (94.5%)	P < 0.0001
Use of intravenous steroids	9 (4.2%)	133 (93.7%)	P < 0.0001
Use of inhaled steroids	11 (7.7%)	131 (92.3%)	P < 0.0001
Hypothyroidism	17 (12.0%)	125 (88.0%)	P < 0.0001
History of prior vitreoretinal surgery	61 (43.0%)	81 (57.0%)	P = 0.095
Ethnicity			P < 0.0001
Arab	125 (88.0%)		
African	10 (7.0%)		
Asian	6 (4.2%)		
European	1 (0.7%)		

Among the patients with ACG, 13 (86.7%) patients had hyperopia. Moreover, a small number of patients with ACG used anticholinergic drugs (13.3%), used sympathomimetics (20.0%), used decongestants (13.3%), and experienced stress/fear (20.0%). There was no significant difference between patients with ACG in terms of age > 60 years, female sex, a history of eye injury, and rubeosis iridis (P > 0.050). Most patients with ACG (80.0%) were of Arabic ethnicity (P = 0.001, Table 3).

**Table 3:** Risk factors for angle-closure glaucoma (n = 15)

Risk factors	Yes	No	Significance
Age > 60 years	10 (66.7%)	5 (33.3%)	P = 0.197
Female sex	8 (53.3%)	7 (46.7%)	P = 0.796
History of eye injury	6 (40.0%)	9 (60.0%)	P = 0.439
Rubeosis iridis	8 (53.3%)	7 (46.7%)	P = 0.796
Hyperopia	13 (86.7%)	2 (13.3%)	P = 0.005
Use of anticholinergic drugs	2 (13.3%)	13 (86.7%)	P = 0.005
Use of sympathomimetics	3 (20.0%)	12 (80.0%)	P = 0.020
Use of decongestants	2 (13.3%)	13 (86.7%)	P = 0.005
Experience of stress/fear	3 (20.0%)	12 (80.0%)	P = 0.020
Ethnicity			P = 0.001
Arab	12 (80.0%)		
African	2 (13.3%)		
Asian	1 (6.7%)		

Among the patients with congenital glaucoma, some patients had a family history of congenital glaucoma (36.2%), history of eye trauma (5.2%), history of eye infection (12.1%), positive history of tumor (1.7%), congenital ocular abnormalities (20.7%), and systemic congenital anomalies (8.5%). There was no significant difference between patients with congenital glaucoma in terms of parental consanguinity (P > 0.050). Most patients with congenital glaucoma were of Arabic ethnicity (95.8%; P < 0.0001, Table 4).

**Table 4:** Risk factors for congenital glaucoma (n = 58)

Risk factors	Yes	No	Significance
Family history of congenital glaucoma	21 (36.2%)	37 (63.8%)	P = 0.036
Consanguineous parents	28 (48.3%)	30 (51.7%)	P = 0.793
History of eyes trauma	3 (5.2%)	55 (94.8%)	P < 0.0001
History of eyes infection	7 (12.1%)	51 (87.9%)	P < 0.0001
Positive history of tumor	1 (1.7%)	57 (98.3%)	P < 0.0001
Congenital ocular abnormalities	12 (20.7%)	46 (79.3%)	P < 0.0001
Systemic congenital abnormalities	5 (8.5%)	53 (91.4%)	P < 0.0001
Ethnicity			P < 0.0001
Arab	55 (95.8%)		
African	3 (5.2%)		

## Discussion

This study examined the prevalence of risk factors among patients with different glaucoma types living in Jeddah, Kingdom of Saudi Arabia. Most patients were aged 19-55 years (n= 54, 25.1%), male (n = 118, 54.9%), and Arab. Most patients had POAG (66.0%), which is consistent with results of previous reports, followed by congenital glaucoma (27.0%) and ACG (7%) [13].

Primary open-angle glaucoma

Several risk factors for POAG were reported [14-18]. We investigated the risk factors for POAG, including age > 40 years; history of high IOP, diabetes mellitus, hypothyroidism, myopia, and family history of glaucoma; history of using topical, oral, intravenous, or inhaled corticosteroids; history of previous vitreoretinal surgery; and ethnicity. The prevalence of POAG dramatically increases with age; accordingly, we found that an age of > 40 years was a significant risk factor for disease onset (122 [85.9%] patients with POAG) [14-18]. IOP elevation (> 21 mmHg) contributes to the onset and progression of POAG [14-19], with both pharmacologic and surgical treatment being directed to lowering IOP [20]. However, we did not observe this relationship among our patients with POAG (high vs. normal IOP: 57.7% vs. 42.3%). Similarly, a Japanese Tajimi study reported that the prevalence of POAG was 3.9%, with up to 90% of the patients having an IOP ≤ 21 mmHg [21]. Furthermore, the Baltimore Eye Survey reported that approximately half of all patients with newly diagnosed POAG had an initial IOP < 21 mmHg [22]. In our study, myopia was significantly associated with the presence of POAG, which is consistent with previous reports that myopia is a significant risk factor for POAG [16-18]. Several hypotheses have been proposed to explain the relationship between diabetes mellitus and POAG [23]. A previous meta-analysis reported that patients with diabetes have a significantly increased risk of developing POAG [24]. However, in our study, only about half (n = 80 [56.3%]) of the patients with POAG had a history of diabetes. Moreover, only 30 (21.1%) patients with POAG had a family history of glaucoma in first-degree relatives, which is inconsistent with findings of previous reports that it is a genetic risk factor for POAG development [14,15,25].

Congenital glaucoma

In this study, we investigated risk factors for congenital glaucoma, including a family history of congenital glaucoma, consanguinity, history of eye trauma, uveitis, tumors, congenital ocular abnormalities, systemic congenital abnormalities, and ethnicity. A positive first-degree family history is often present in patients with congenital glaucoma through either autosomal dominant/recessive inheritance [26]. In our study, 21 (36.2%) patients had a positive first-degree family history and 28 (48.3%) patients had consanguine parents, which could be attributed to the low awareness level of disease inheritance and reluctance to seek genetic counseling.

Angle-closure glaucoma

In our study, ACG was the least common glaucoma type. We investigated the risk factors for ACG, including age > 60 years, female sex, a history of eye injury, rubeosis iridis, hyperopia, anticholinergic drug use, sympathomimetic use, decongestant use, experience of stress/fear, and ethnicity. The prevalence of ACG increases with age [27]. Accordingly, 66.7% of our patients with ACG were aged > 60 years. Furthermore, the predominant sex was female, with no significant between-sex difference, which is consistent with previous reports [28-30]. The anatomic eye features are crucially involved in ACG development. A shallow anterior chamber is an established risk factor for ACG development, with 86.7% of our patients with ACG showing a short axial length of the eye [31]. Although there have been numerous reports of drug-induced glaucoma, we observed a significantly low prevalence of patients who used anticholinergic, sympathomimetic, and decongestant drugs that induced ACG. This finding could be attributed to our low sample size. Cohen and Hajoff examined 52 patients in a randomized study, focusing on a significant life event assessed by two psychiatrists, blinded to the patient's eye condition [32]. There were emotional events in 13 patients with glaucoma and six healthy controls. However, in our study, only three (20%) patients with ACG had experienced stress [32].

Limitations and recommendations

This study has several limitations. First, this was a small-scale single-center study, which limits the generalizability of our findings. Second, most participants were of Arabic ethnicity since this study was conducted in an Arabic country. Finally, since we included a small number of patients with ACG, our findings cannot conclusively determine its risk factors. Further large-scale multicenter studies in Saudi Arabia are warranted to address this gap.

## Conclusions

Our findings demonstrated that POAG was the most common glaucoma subtype, followed by congenital glaucoma and ACG. The most common risk factor for POAG was age > 40 years, followed by myopia. Moreover, most participants with congenital glaucoma had a history of consanguine parents and a positive family history of congenital glaucoma. Regarding ACG, the most common risk factors were hyperopia, advanced age, and female sex, in this order. Most participants were of Arabic ethnicity since this study was conducted in an Arabic country. These findings could inform public health policies among practitioners involved in ophthalmological care.

## References

[1] Leffler CT, Schwartz SG, Hadi TM, Salman A, Vasuki V (2015). The early history of glaucoma: the glaucous eye (800 BC to 1050 AD). Clin Ophthalmol.

[2] Ivanišević M (2019). First look into the eye. Eur J Ophthalmol.

[3] Keeler CR (2002). The ophthalmoscope in the lifetime of Hermann von Helmholtz. Arch Ophthalmol.

[4] Leffler CT, Schwartz SG, Giliberti FM, Young MT, Bermudez D (2015). What was glaucoma called before the 20th century?. Ophthalmol Eye Dis.

[5] Casson RJ, Chidlow G, Wood JP, Crowston JG, Goldberg I (2012). Definition of glaucoma: clinical and experimental concepts. Clin Exp Ophthalmol.

[6] Kingman S (2004). Glaucoma is second leading cause of blindness globally. Bull World Health Organ.

[7] Boyd K (2022). What is glaucoma? symptoms, causes, diagnosis, treatment [Internet]. What Is glaucoma? Symptoms, causes, diagnosis, treatment.. https://www.aao.org/eye-health/diseases/what-is-glaucoma.

[8] Distelhorst JS, Hughes GM (2003). Open-angle glaucoma. Am Fam Physician.

[9] Francis BA and Khondkaryan A (2022). Angle-closure glaucoma. https://www.aao.org/education/current-insight/angleclosure-glaucoma-19.

[10] Jain V, Jain M, Abdull MM, Bastawrous A (2017). The association between cigarette smoking and primary open-angle glaucoma: a systematic review. Int Ophthalmol.

[11] Khandekar R, Chauhan D, Yasir ZH, Al-Zobidi M, Judaibi R, Edward DP (2019). The prevalence and determinants of glaucoma among 40 years and older Saudi residents in the Riyadh Governorate (except the Capital) - a community based survey. Saudi J Ophthalmol.

[12] Tham YC, Li X, Wong TY, Quigley HA, Aung T, Cheng CY (2014). Global prevalence of glaucoma and projections of glaucoma burden through 2040: a systematic review and meta-analysis. Ophthalmology.

[13] Mahabadi N, Foris LA, Tripathy K (2023). Open angle glaucoma. StatPearls [Internet].

[14] Le A, Mukesh BN, McCarty CA, Taylor HR (2003). Risk factors associated with the incidence of open-angle glaucoma: the visual impairment project. Invest Ophthalmol Vis Sci.

[15] Leske MC, Wu SY, Hennis A, Honkanen R, Nemesure B (2008). Risk factors for incident open-angle glaucoma: the Barbados Eye Studies. Ophthalmology.

[16] Pan CW, Yang WY, Hu DN (2017). Longitudinal cohort study on the incidence of primary open-angle glaucoma in Bai Chinese. Am J Ophthalmol.

[17] Sia DI, Edussuriya K, Sennanayake S, Senaratne T, Selva D, Casson RJ (2010). Prevalence of and risk factors for primary open-angle glaucoma in central Sri Lanka: the Kandy eye study. Ophthalmic Epidemiol.

[18] Suzuki Y, Iwase A, Araie M (2006). Risk factors for open-angle glaucoma in a Japanese population: the Tajimi Study. Ophthalmology.

[19] Coleman AL, Miglior S (2008). Risk factors for glaucoma onset and progression. Surv Ophthalmol.

[20] Kass MA, Heuer DK, Higginbotham EJ (2002). The ocular hypertension treatment study: a randomized trial determines that topical ocular hypotensive medication delays or prevents the onset of primary open-angle glaucoma. Arch Ophthalmol.

[21] Iwase A, Suzuki Y, Araie M (2004). The prevalence of primary open-angle glaucoma in Japanese: the Tajimi Study. Ophthalmology.

[22] Sommer A, Tielsch JM, Katz J, Quigley HA, Gottsch JD, Javitt J, Singh K (1991). Relationship between intraocular pressure and primary open angle glaucoma among white and black Americans. The Baltimore Eye Survey. Arch Ophthalmol.

[23] Shen L, Walter S, Melles RB, Glymour MM, Jorgenson E (2016). Diabetes pathology and risk of primary open-angle glaucoma: evaluating causal mechanisms by using genetic information. Am J Epidemiol.

[24] Zhao YX, Chen XW (2017). Diabetes and risk of glaucoma: systematic review and a meta-analysis of prospective cohort studies. Int J Ophthalmol.

[25] Kaimbo DK, Buntinx F, Missotten L (2001). Risk factors for open-angle glaucoma: a case-control study. J Clin Epidemiol.

[26] Abu-Amero KK, Edward DP (2004). Primary congenital glaucoma. GeneReviews® [Internet].

[27] Seah SK, Foster PJ, Chew PT, Jap A, Oen F, Fam HB, Lim AS (1997). Incidence of acute primary angle-closure glaucoma in Singapore. An island-wide survey. Arch Ophthalmol.

[28] Bourne RR, Sukudom P, Foster PJ (2003). Prevalence of glaucoma in Thailand: a population based survey in Rom Klao District, Bangkok. Br J Ophthalmol.

[29] Foster PJ, Oen FT, Machin D (2000). The prevalence of glaucoma in Chinese residents of Singapore: a cross-sectional population survey of the Tanjong Pagar district. Arch Ophthalmol.

[30] Vijaya L, George R, Arvind H (2006). Prevalence of angle-closure disease in a rural southern Indian population. Arch Ophthalmol.

[31] Yanoff M, Duker JS (2008). Ophthalmology Third Edition.

[32] Cohen SI, Hajioff J (1972). Life events and the onset of acute closed-angle glaucoma. J Psychosom Res.

